# Supplementary material for: Epidermal growth factor receptor activation is essential for kidney fibrosis development
Source: Nat Commun. 2023 Nov 14;14:7357. doi: 10.1038/s41467-023-43226-x (PMC10645887; doi:10.1038/s41467-023-43226-x)
Supplement: Supplementary file 1 — Supplementary Information [file 41467_2023_43226_MOESM1_ESM.pdf]

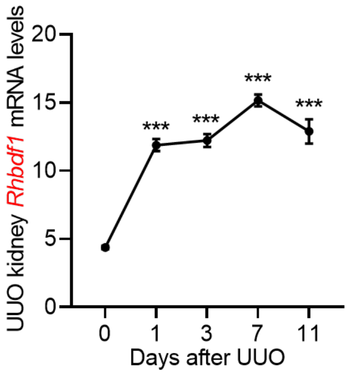

Suppl Figure S1. Kidney iRhom1 mRNA increased progressively following unilateral ureteral obstruction (UUO).

Data are means ± SEM, ****P*<0.001, analyzed using 2-way ANOVA followed by Tukey's post hoc test. n=7 and 8.

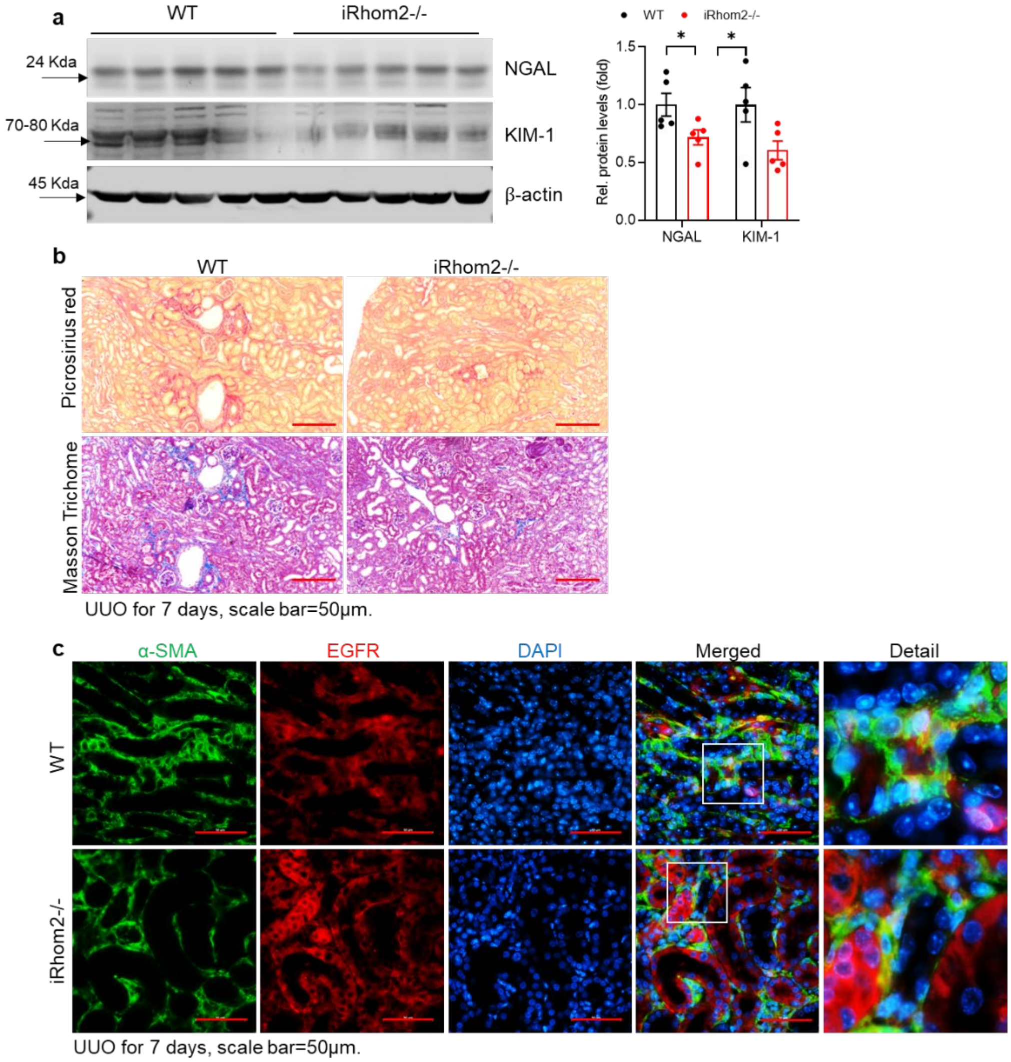

Suppl Figure S2. iRhom2 knockout mice had less kidney injury and fibrosis following unilateral ureteral obstruction (UUO). Both WT and iRhom2-/- mice underwent UUO for 7 days. iRhom2-/- had less tubular injury as indicated by lower NGAL and KIM-1 protein expression (n=5) (**a**) and less kidney fibrosis as indicated by Picrosirius red and Masson trichrome stain (**b**). (**c**) α-SMA and EGFR colocalization was similar in iRhom2-/- mice and WT mice. Scale bar=50µm for all.

Data are means ± SEM, *P<0.05, analyzed using two tailed Student's t test.

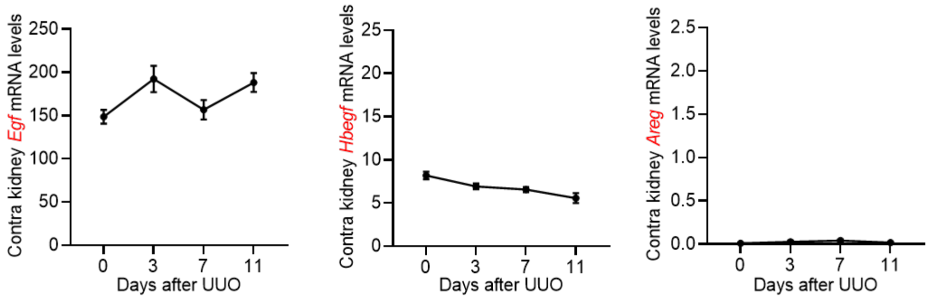

Suppl Figure S3

Suppl Figure S3. EGFR ligands in the non-obstructed contralateral kidney were not increased following unilateral ureteral obstruction (UUO). Kidney mRNA levels of *Egf, Hbegf*, and *Areg* were not increased after UUO. n=6 and 8.

Data are means ± SEM, analyzed using 2-way ANOVA followed by Tukey's post hoc test for all.

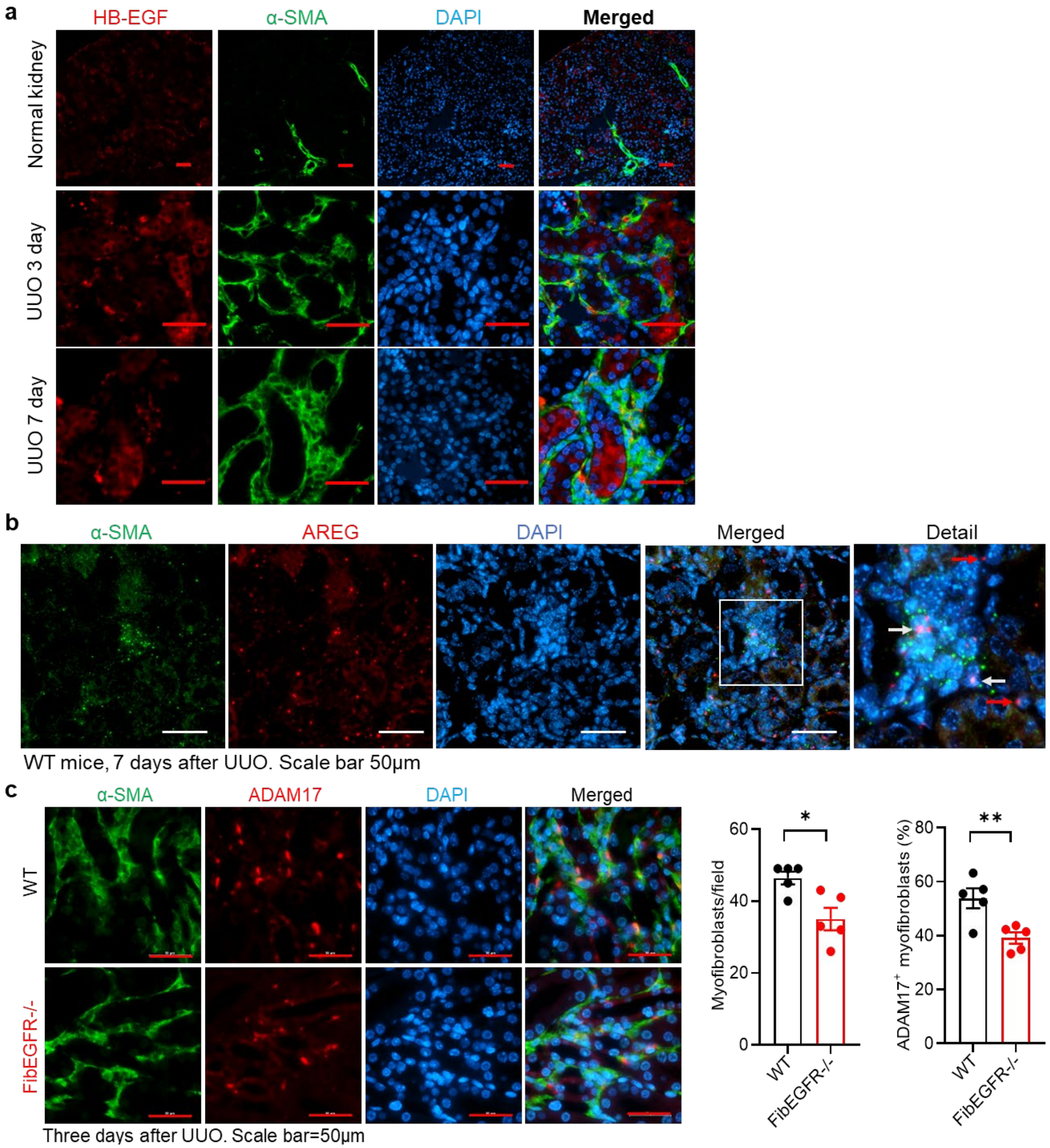

Suppl Figure S4. HB-EGF and AREG and ADAM17 increased following unilateral ureteral obstruction (UUO). Mice underwent UUO for 3 days and 7 days. (**a**) HB-EGF expression was minimal in control kidney but evident in both tubular epithelial cells and myofibroblasts (co-localization with α-SMA) in WT mouse kidney at day 3 and day 7 after UUO. (**b**) RNAscope showed Areg mRNA in tubular epithelial cells (red arrows) and myofibroblasts (while arrows, colocalization with Acta2, which encodes α-SMA). (**c**) Three days after UUO, ADAM17 was primarily localized to myofibroblasts, and its expression was increased in FibEGFR-/- mice. N=5. Scale bar=50μm for all.

Data are means ± SEM, **P*<0.05, ***P*<0.01, analyzed using two tailed Student's t test.

a

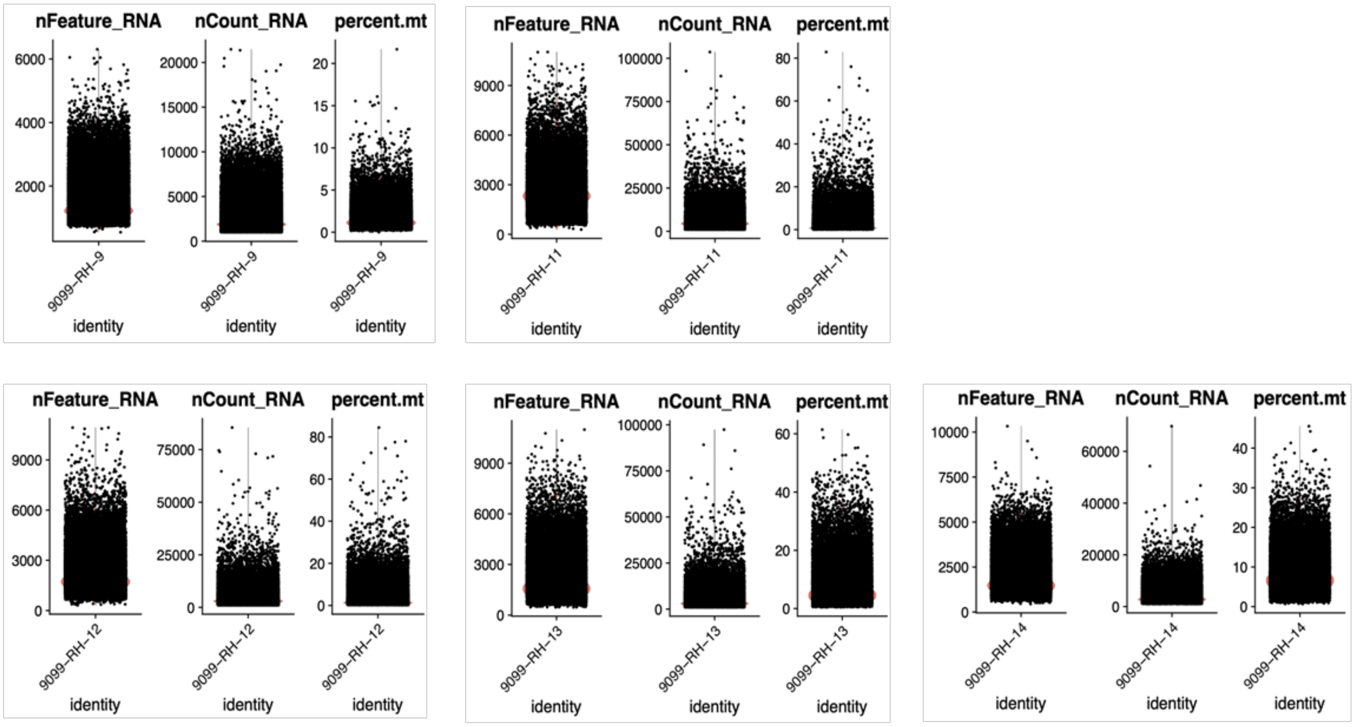

b

Samples	Treatment	cell.count	gene.median	UMI.mean
9099-RH-13	WT, 1d UUO	32151	2010	5071.93683
9099-RH-14	FibEGFR-/-, 1d UUO	42501	1800	4131.50928
9099-RH-9	WT, 3d UUO	47975	1507	2898.14843
9099-RH-11	FibEGFR-/-, 3d UUO	23343	2488	6364.46382
9099-RH-12	FibEGFR-/-, 3d UUO	36634	2043	4796.5796

Note: 9099-RH-11 and 12 were from the sample and combined for further analysis.

Suppl Figure S5. Single-nucleus RNA sequencing (snRNAseq) analysis of fibrotic kidneys. Kidneys from both WT mice and FibEGFR-/- mice at day 1 and day 3 after UUO were analyzed via Vanderbilt VANTAGE NGS sequencing core. Both cell counts and gene numbers were acceptable for further analysis.

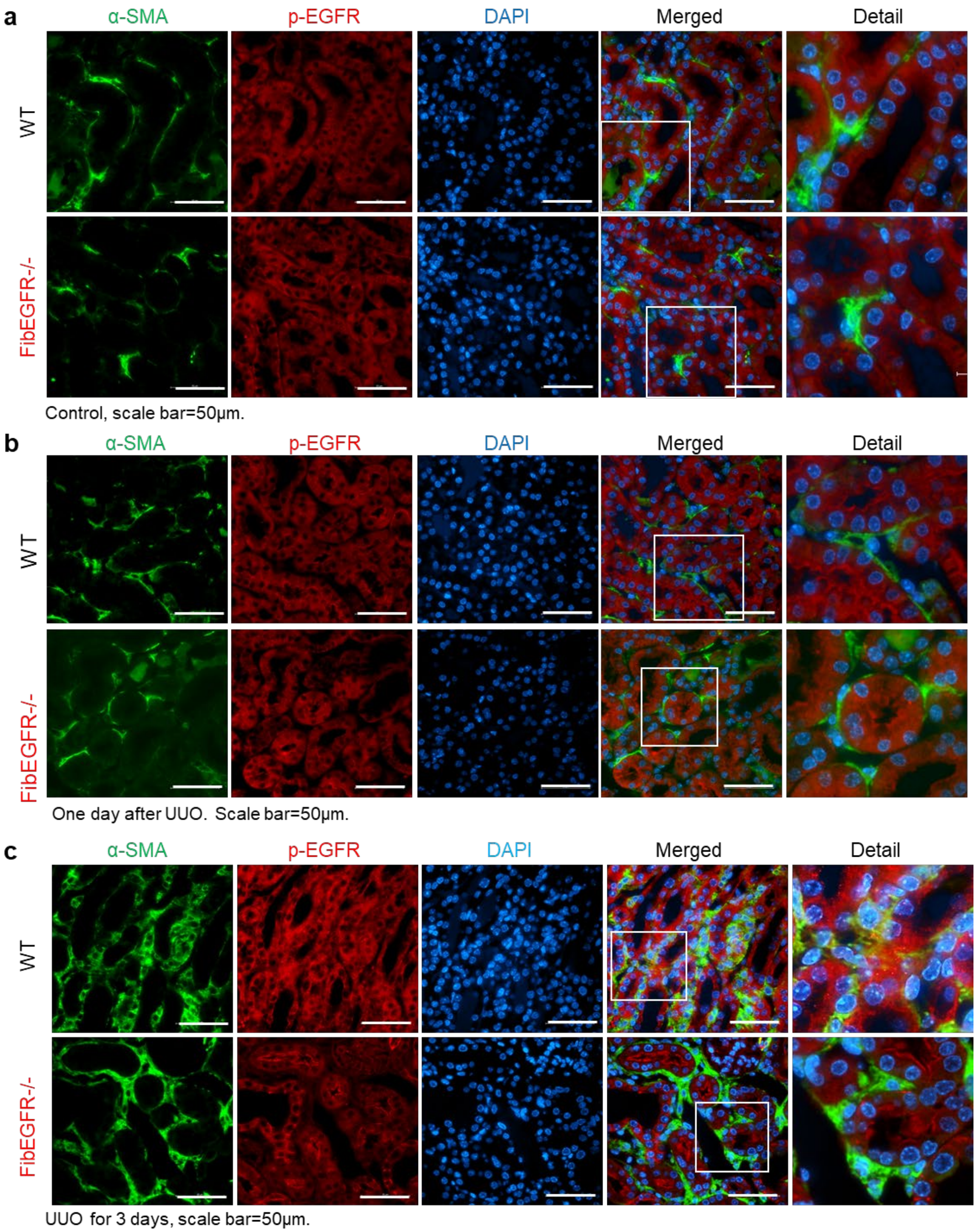

Suppl Figure S6. Mice with selective fibroblast EGFR deletion had minimal p-EGFR expression in myofibroblasts following unilateral ureteral obstruction (UUO). Both WT and FibEGFR-/- mice underwent UUO for 1 and 3 days. p-EGFR expression, a marker of EGFR activation, was minimal in α-SMA+ cells in WT and FibEGFR-/- kidneys at day 0 (**a**) and day 1 (**b**) after UUO. (**c**) Apparent p-EGFR colocalization with α-SMA+ cells was observed in WT mice but not FibEGFR-/- mice at 3 days after UUO. Scale bar=50μm for all.

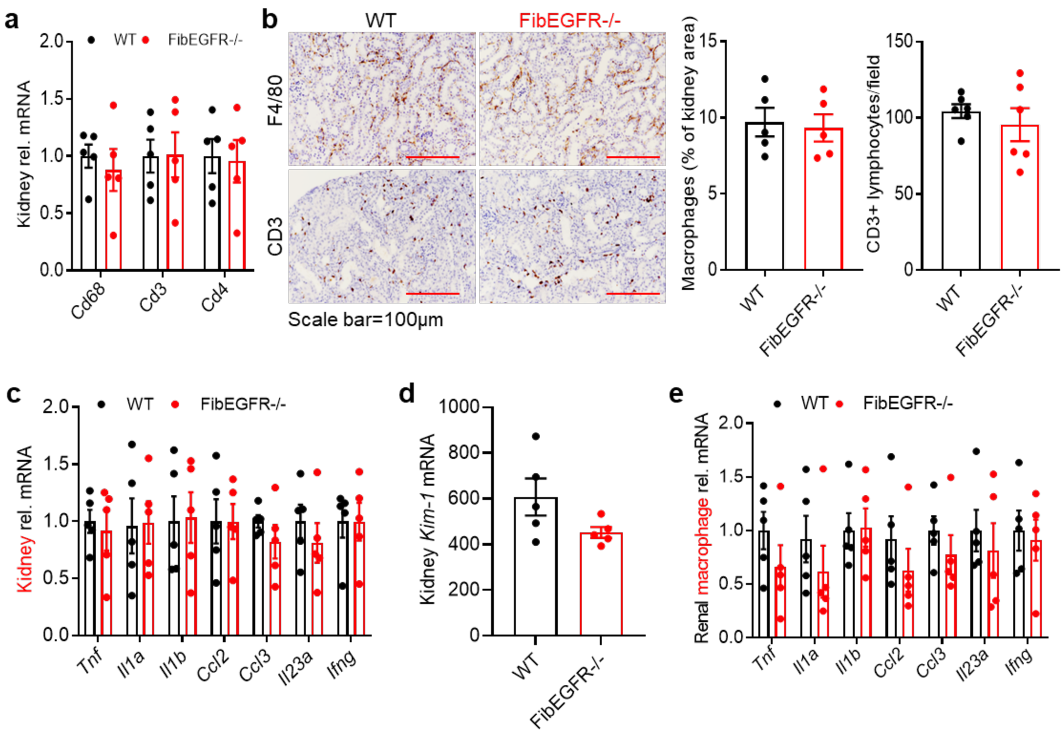

Suppl Figure S7. Fibroblast EGFR deficiency did not affect immune cell infiltration after unilateral ureteral obstruction (UUO). WT mice and FibEGFR-/- underwent UUO for 7 days. (**A** and **B**) Similar kidney immune cell infiltration was observed in FibEGFR-/- and WT mice after UUO as indicated by similar transcripts of *Cd68*, *Cd3*, and *Cd4* (n=5) (**a**) as well as similar macrophage and lymphocyte density in the kidneys (n=5) (**b**). Scale bar=100μm. Similar kidney transcripts of proinflammatory cytokines/chemokines including *Tnf*, *Il1a*, *Il1b*, *Ccl2*, *Ccl3*, *Il23a* and *Ifng* (**c**) and kidney injury (*Kim-1*) (**d**) were observed in FibEGFR-/- and WT mice after UUO. n=5. (**e**) Similar transcripts of kidney macrophage proinflammatory cytokines/chemokines were also observed in FibEGFR-/- and WT mice after UUO. n=5.

Data are means ± SEM, analyzed using two tailed Student's t test for all.

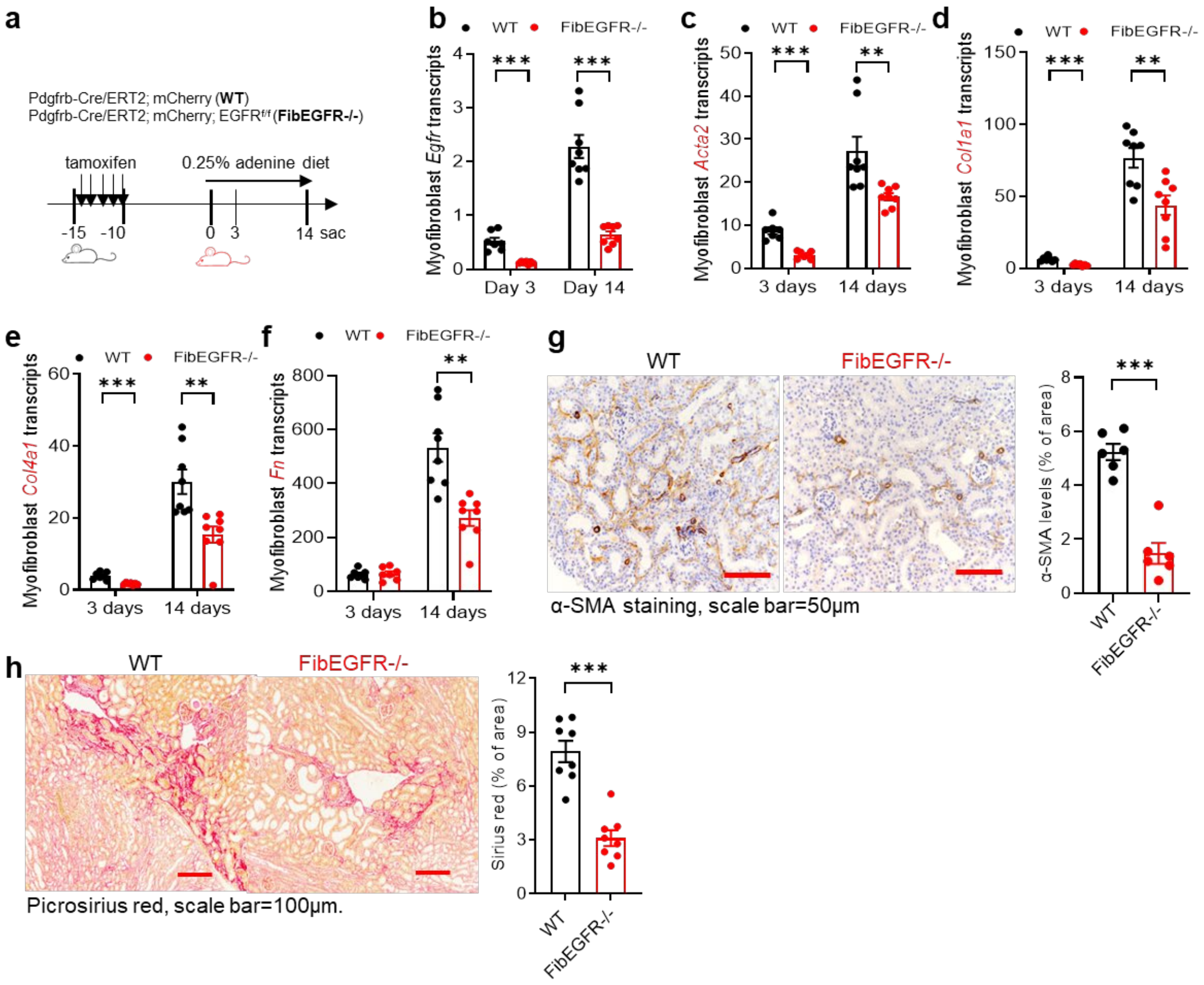

Suppl Figure S8. Fibroblast EGFR deficiency attenuated kidney fibrosis in adenine nephropathy. (**a**) Schematic of experimental protocol. In isolated kidney myofibroblasts at day 3 and day 14 on adenine diet, the transcripts of *Egfr* (**b**), *Acta2* (**c**), *Col1a1* (c), *Col4a1* (**e**), and *Fn* (**f**) were lower in FibEGFR-/- mice than WT mice. n=7 and 8. FibEGFR-/- mice developed less kidney fibrosis at day 14 on adenine diet, as indicated by quantitative α-SMA immunostaining (n=6) (**g**) and Picrosirius red staining (n=8) (**h**).

Data are means ± SEM, ***P*<0.01, ****P*<0.001, analyzed using two tailed Student's t test for all.

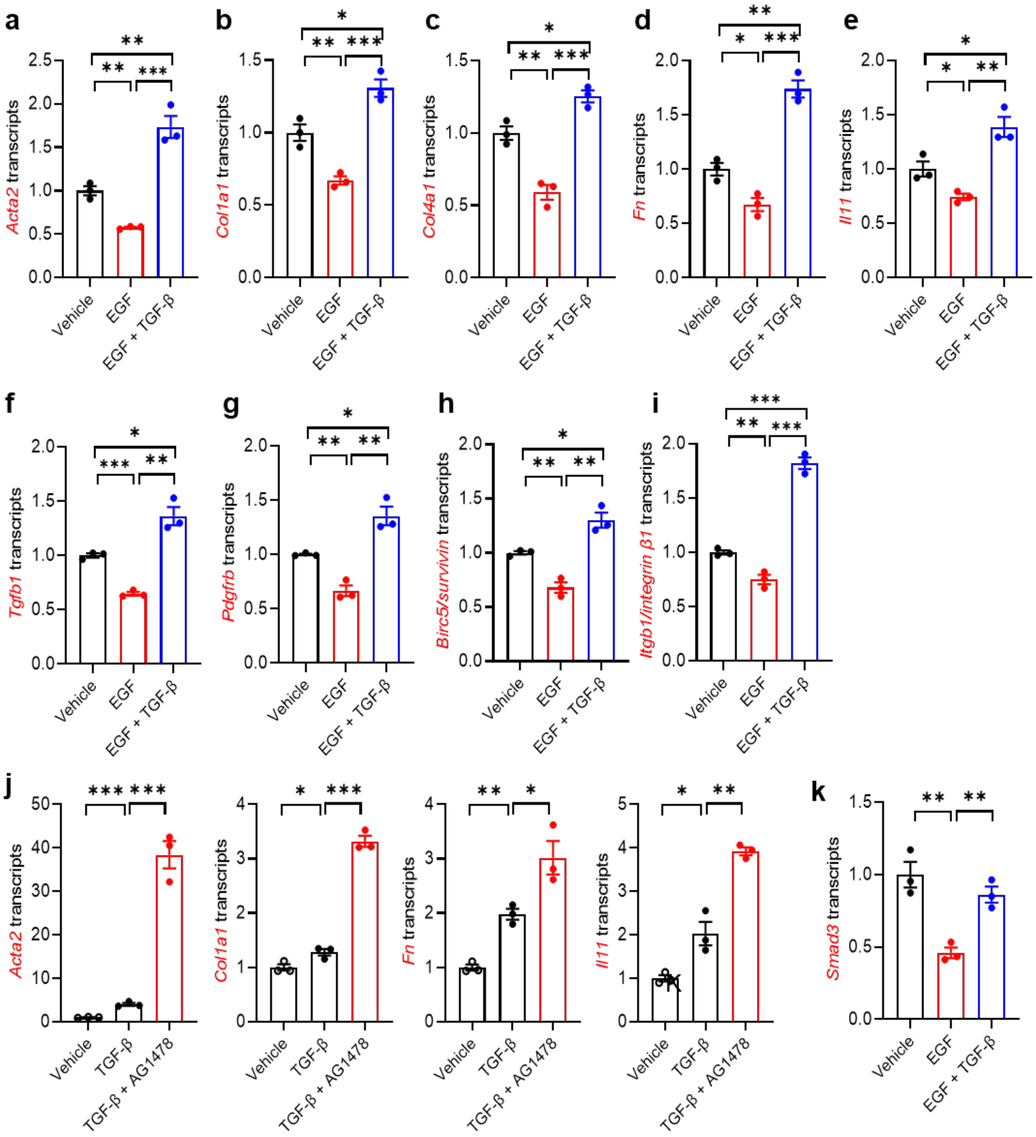

Suppl Figure S9. **TGF-β signaling overcame EGFR mediated inhibition of myofibroblast differentiation.** Fibroblasts were treated with EGF with or without TGF-β for 48 h as described in Methods. (**a-i**) EGF-mediated inhibition of mRNA levels of profibrotic and fibrotic genes were reversed and further stimulated by TGF-β. n=3. (**j**) Inhibition of EGFR tyrosine kinase activity with AG1478 augmented TGF-β-induced *Acta2*, *Col1a1*, *Fn*, and *Il11* expression. n=3. (**k**) EGF inhibition of *Smad3* expression was reversed by coadministration of TGF-β. n=3.

Data are means ± SEM, **P*<0.05, ***P*<0.01, ****P*<0.001, analyzed using 2-way ANOVA followed by Bonferroni's post hoc test for all.

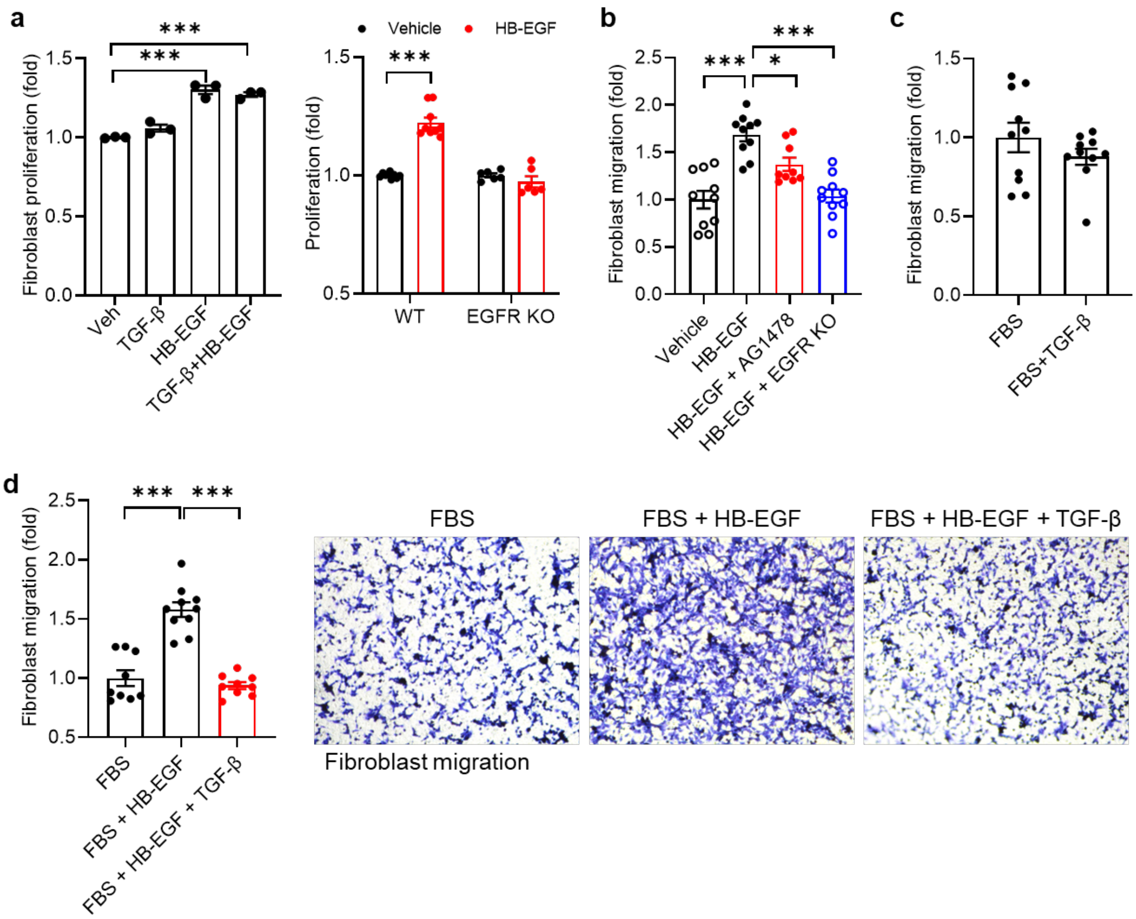

Suppl Figure S10. EGFR activation stimulated fibroblast proliferation and migration and TGF-β antagonized EGFR-mediated migration. Mouse fibroblast proliferation was evaluated with flow cytometry of EdU incorporation and migration with Boyden Chamber assay as described in Methods. (**a**) HB-EGF stimulated proliferation in WT but not in EGFR knockout fibroblasts. TGF-β had no effect on HB-EGF-induced proliferation. n=3. (**b**) HB-EGF-induced fibroblast migration was attenuated by EGFR inhibition with AG1478 or EGFR knockout. n=3. TGF-β alone had no effect on fibroblast migration (**c**) but inhibited HB-EGF-induced migration (**d**). n=3.

Data are means ± SEM, **P*<0.05, ****P*<0.001, analyzed using 2-way ANOVA followed by Bonferroni's post hoc test for all.

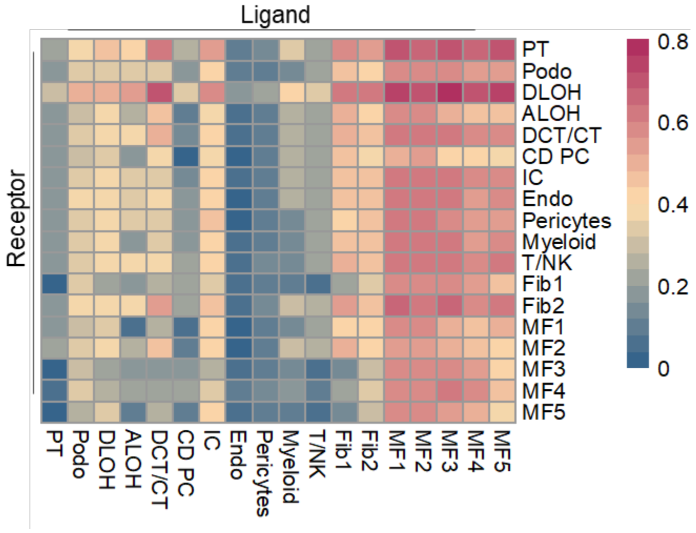

Suppl Figure S11. snRNAseq analysis shows intercellular crosstalk between the fibroblasts/myofibroblasts and other cell types. Ligands and receptors with enrichment were paired to elucidate intercellular signal transduction networks. All five Myofibroblast clusters had increased gene expression for ligands that can theoretically interact with receptors on kidney epithelium, endothelium, pericytes and innate and adaptive immune cells.

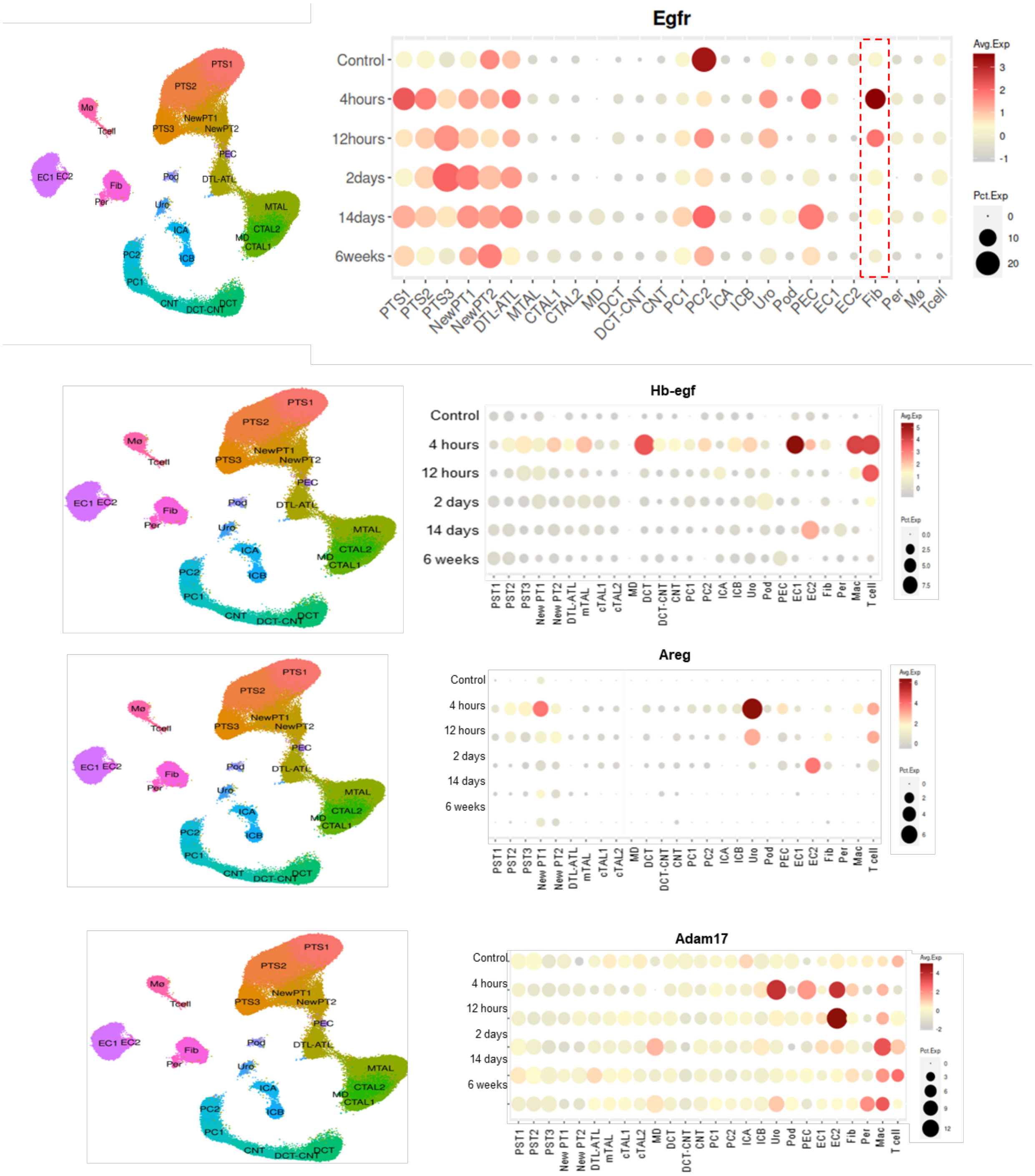

Suppl Figure S12. Re-analysis of published mouse kidney snRNAseq datasets from ischemic reperfusion injury by Humphreys group[34] indicate increased EGFR mRNA levels in fibroblasts and other cell types as well as increased mRNA levels of HB-EGF and AREG and ADAM17 after ischemic injury.

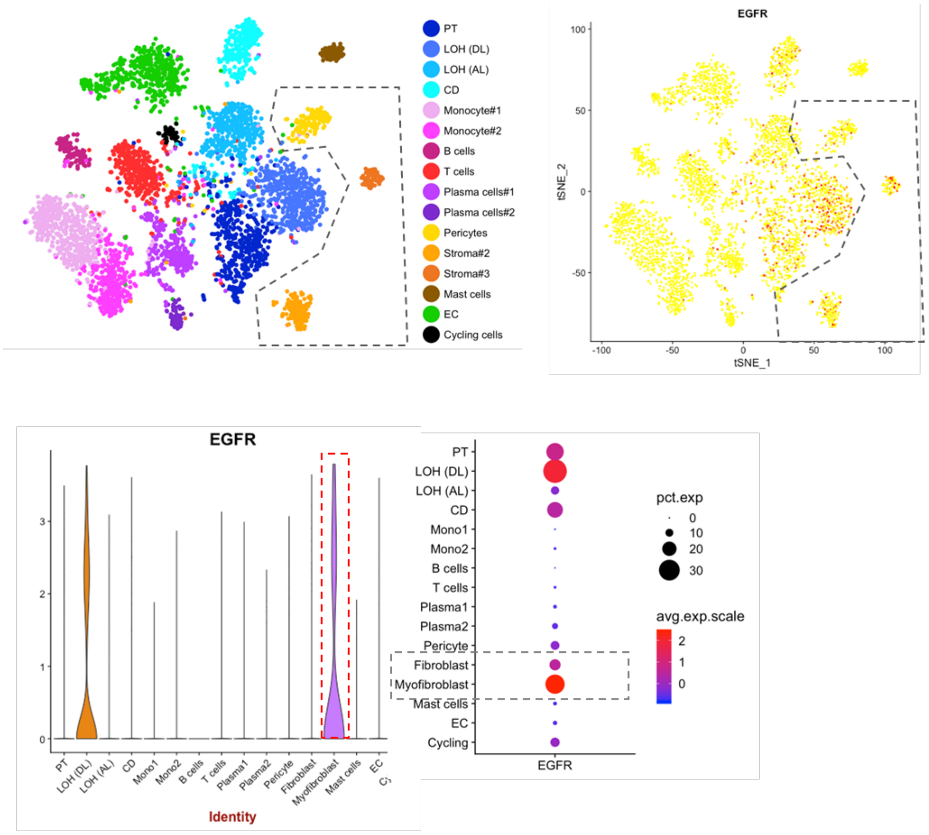

Suppl Figure S13. Re-analysis of published snRNAseq datasets from allograft kidney biopsy by Humphreys group[43] indicate increased EGFR mRNA levels in fibroblasts and myofibroblasts among others.

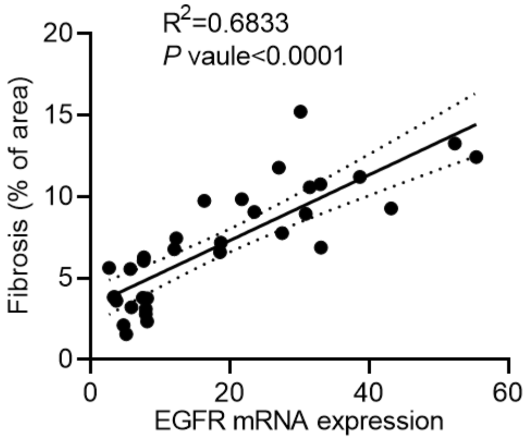

Suppl Figure S14. Single linear regression analysis shows positive correlation between EGFR mRNA levels in myofibroblasts and kidney fibrosis levels (n=33, a combination of data from mice with treatment of 7 day UUO, 19 day folic acid and 14 day adenine).

Table S1. List of primers for genotyping.

Alleles/Transgenes	Primer
EGFR-flox	EGFR-S: 5′-CTTTGGAGAACCTGCAGATC-3′<br>EGFR-AS: 5′-CTGCTACTGGCTCAAGTTTC-3′
mCherry-flox	10507: 5′-TTATGTAACGCGGAACTCCA-3′<br>oIMR 8545: 5′-AAAGTCGCTCTGAGTTGTTAT-3′<br>Oimr8546: 5′-GGAGCGGGAGAAATGGATATG-3′
PDGFRb-Cre	15495: 5′-ACATGTCCATCAGGTTCTTGC-3′<br>36182: 5′-CCACCTTGAATGAAGTCAACAC-3′<br>36183: 5′-AGCTTGTGGCAGTGTAGCTG-3′
iRhom2-/-	F: 5′-CTCTCTCTCTGTCTCTCTCTCT-3′<br>R: 5′-TCTTAACGGCTGAGCCATTGCTC-3′

Table S2. List of antibody, source, application, and dilution

Antibody	Catalog Number	Dilution
Rabbit anti-EGFR (Y1068)	Abcam, ab40815	IF 1:50
Goat anti-Collagen I	SouthernBiotech, 1310-01	WB 1:500
Rabbit-anti-Fibronectin	Sigma-Aldrich, F3648	WB 1:1000
Mouse anti-α-SMA	Sigma-Aldrich, A5228	WB 1:2000, IF1:300
Rabbit anti-Col IV	Rockland, 6004011060.1	WB 1:500
Rabbit anti-RHBDF2	Abcepta, AP13588A	IF 1:100
Rabbit anti-CD31	Novus, NB100-2284	IF 1:100
Rat anti-CD140b (PDGFRB)	ThermoFisher, 25140282	IF 1:100
Mouse anti-α-Tubulin	Cell signal technology, 3873	WB 1:2000
Rabbit anti-Ki67	Abcam, ab16667	IF 1:300
Rabbit anti-pEGFR	Santa Cruz, SC-12351	WB 1:500
Rabbit anti-pSmad3	ThermoFisher, 600401919	IF 1:100
Rat anti-F4/80	BioRad, MCA497	IHC 1:100
Anti-mouse CD45 (30-F11)	Biolegend 103149	FC 0.2 mg/ml
Anti-mouse CD31 (390)	Biolegend 102440	FC 0.2 mg/ml
Anti-mouse CD140b (APB5)	Biolegend 136006	FC 0.2 mg/ml
Rat-anti CD3	MCA1477	

Table S3. List of gene probes (Applied Biosystems)

Mouse primer	Catalog Number
Emr1	Mm00802529
Cd68	Mm03047343
Tnf	Mm99999068
Il1b	Mm00434228
Il6	Mm00446190
Il11	Mm00434162
Col1a1	Mm00801666
Col3a1	Mm01254476
Fn1	Mm01256744
Col4a1	Mm01210125
Acta2	Mm01546133
Tgfb1	Mm00441726
Tgfb2	Mm00436955
Tgfb3	Mm00436960
Havcr1	Mm00506686
CD3	Mm00442746
CD4	Mm00442754
CD8a	Mm01182107
Il1α	Mm00439621
Pdgfrb	Mm00435553
Birc5	Mm00599749
Itgb1	Mm01253230
Smad3	Mm01170760
Ctgf	Mm01192933
Mrc1	Mm01329362
Arg1	Mm00475991
Ccl2	Mm00441242

Ccl3	Mm00441258
Egfr	Mm00433023
Tgfa	Mm00446232
Hb-egf	Mm00439307
Areg	Mm00437583
Btc	Mm00432137
Ereg	Mm00514794
Rhbdf2	Mm00553470
Rhbdf1	Mm00711711
Adam17	Mm00456428
Adam10	Mm00545742
Epgn	Mm00504344
Acta2	Mm01546133
Il23a	Mm00518984
Ifng	Mm01168134
